# Most Effective Interventions for Enhancing Neural Plasticity for Functional Recovery in Patients with Traumatic Brain Injury

**DOI:** 10.26502/jsr.10020479

**Published:** 2025-11-17

**Authors:** Ambria Pogue, Devendra K. Agrawal

**Affiliations:** Department of Translational Research, College of Osteopathic Medicine of the Pacific, Western University of Health Sciences, Pomona, California 91766 USA

**Keywords:** Compensatory cognitive training with supported employment, Functional recovery, Glasgow Coma Scale, Multidisciplinary rehabilitation, Neuroinflammation, Neuromodulation, Neuroplasticity, Outcome measures, Regenerative medicine, Traumatic brain injury

## Abstract

Traumatic brain injury (TBI) is a major global health concern and one of the leading causes of disability worldwide. Outcomes of this injury range from full recovery to lifelong cognitive, physical, and psychological impairments. Management of TBI remains challenging due to the variety of symptoms and severity, as well as the complexity of the underlying cellular mechanisms. In addition, the lack of standardized treatment protocols contributes to the uncertainty of recovery. This article explores current evidence of the most effective interventions in improving function in patients with TBI. Acute management focuses on patient stabilization, tight control of intracranial pressure, and seizure prophylaxis. Long-term recovery, however, is dependent on a multidisciplinary approach including compensatory cognitive training with supported employment and social communication therapy, which have been shown to accelerate return to work and social reintegration. In addition, structured exercise, dietary modifications, and environmental enrichment have proven to be helpful. Novel therapeutic therapies including neuromodulation techniques and regenerative medicine strategies show promise in enhancing neuroplasticity and repairing injured neural tissue. Although encouraging, these treatments remain experimental, and no FDA approved disease modifying agents exist currently. Future progress will likely center around advancing these novel interventions as well as developing reliable biomarkers to help assess injury severity, predict prognosis, and guide individualized treatment planning.

## Introduction

Traumatic brain injury (TBI) can be a result of a direct impact to the head, penetrating injuries, or rapid acceleration and deceleration injuries. Thus, the risk factors and high-risk groups are closely tied to the causes of the injury. Groups at increased risk include older adults, athletes in contact sports, military personnel, and individuals involved in motor vehicle accidents [1]. Older adults experience the highest rates of TBI compared to any other age group. This increased vulnerability is due to age-related factors such as impaired balance and strength, polypharmacy, and visual deficits, all of which elevate the risk of falls. Notably, approximately 80% of mild TBIs in older adults are caused by falls [2]. On the other end of the age spectrum, children and teenagers are also at an elevated risk due to high levels of physical activity and participation in contact sports. Sports such as football, hockey, boxing and soccer often involve direct head impacts. Young athletes are especially susceptible to repeated concussions, which are associated with both acute TBIs and the potential long-term development of chronic traumatic encephalopathy (CTE) [3]. Military personnel face unique risks, including blast-related injuries and training accidents. According to the U.S. Department of Defense, between 2000 and 2021, approximately 450,000 service members sustained a TBI, with 82% classified as mild [4]. Motor vehicle accidents remain a common cause of TBI, with risk further increased by factors such as alcohol use while driving and adolescent men [5]. Other factors contributing to the risk of TBI include low socioeconomic status, substance use, and high-risk occupations such as transportation, construction, commercial fishing, or mining [1].

### Clinical Symptoms

The symptoms associated with TBI vary in both the severity and length of time. Symptoms include headache, loss of consciousness, nausea, vomiting, cognitive impairment, balance problems, sleep disturbances and many more. Loss of consciousness, nausea, and vomiting are often seen in the acute phase of TBI and can help predict the severity of the injury. Headache can be present in both the acute and chronic phase. In the acute phase, headache can be due to direct injury, whereas in the chronic phase it is often from post-concussive syndrome. Direct injury can also cause disruption to the neural circuits, resulting in memory impairments or cognitive slowing. These symptoms can arise immediately and last from several days to months, depending on the severity of the injury. Fatigue and concentration difficulties are cited among the most disruptive of symptoms following TBI and is also thought to be one of the longest lasting symptoms. Sleep disturbances are often tied to the severity of TBI. Mild TBI is often associated with insomnia due to disruption of the circadian rhythm, whereas more severe injury often results in hypersomnolence disorders. Balance problems and spasticity are often associated with severe TBI and can have a significant impact on a patient’s ability to return to activities of daily living [6]. Psychologic symptoms are also common following TBI, including depression, impulsivity, and anxiety. Depression can occur in up to 50% of adults who have had a moderate to severe TBI. Studies have shown that pharmacologic control of these neuropsychiatric symptoms can often improve symptoms in other domains [7]. All the symptoms listed above are commonly experienced by those with TBI. However, there are other potential less common, more serious consequences of this type of injury. One such concern is the increased risk of stroke among patients who have suffered from a moderate to severe TBI [8]. Another concern is the risk of Alzheimer’s disease in patients who have sustained TBI. Although some studies show the risk of Alzheimer’s is higher in military patients who have sustained TBI, other studies conclude that there is no association [9].

### Evaluation of Severity

Closely tied to the symptoms a patient experiences are the severity of the injury. TBI severity is classified in several different ways. One strategy is using the Glasgow Coma Scale Score (GCS) which uses eye opening response, verbal response, and motor response on a scale of 3–15 to assess neurologic deficit. Mild TBI is often classified of GCS 13–15, moderate 9–12, and severe 3–8 [10]. Although this scale, developed in 1974, is an efficient tool in an emergency setting, it lacks prognostic ability and discounts several factors that aid in determining the level of consciousness of the patients. Interestingly, several studies have demonstrated that the individual components of the GCS are more diagnostic and predictive of outcomes when looked at individually than when pooled together into one generic score [11]. The GCS is often used in conjunction with other predictive and diagnostic scales. Another such tool is determining the time of post traumatic amnesia (PTA). PTA can be assessed based on two different scales, one of which includes both anterograde and retrograde amnesia (GOAT, Galveston Orientation and Amnesia Test) and the other which only includes anterograde amnesia (WPTAS, Westmead PTA Scale). Although these two scales can differ in the length of time necessary to classify a severe TBI, greater than one week of PTA usually satisfies this requirement [12]. Although this system accounts for some aspects the GCS does not account for, it is not without its limitations. One study suggests that PTA does not account for confounders, such as use of opiates in association with TBI, which can lead to a falsely increased PTA resulting in misclassification of TBI [12]. Another way to determine severity of TBI includes determining if there was loss of consciousness (LOC) or any acute neuroimaging findings. Neuroimaging can be helpful in moderate to severe cases of TBI, whereas mild cases typically do not have any imaging findings. In TBI with significant damage, it is possible to see skull fractures or acute bleeding via routine imaging such as CT or MRI [13]. LOC is often looked at in a timeframe following the injury. If there is no LOC or less than 30 minutes of LOC, then TBI is generally classified as mild. Greater than 24 hours of LOC often correlates to severe TBI. These diagnostic tools are summarized in table 1.

A more recent addition to the evaluation of TBI is looking at disorders of consciousness (DoC). DoC can be classified as coma, vegetative state/unwakefulness state, minimally conscious state (MCS), and post-traumatic confusional state. Minimally conscious state can further be divided into MCS+ or MCS− depending on whether language function is present or absent, respectively. These states have been studied in relation to GCS, which confirmed that GCS does not reflect the level of consciousness accurately, which is an important factor when diagnosing and predicting outcomes of GCS [11]. In addition to evaluating immediate injury, scales have also been developed to determine the outcome of the injury. One such scale is known as the Glascow Outcome Scale-Extended (GOSE) which will be discussed in more detail when exploring prognosis.

### Epidemiology

An estimated 64–74 million people sustain a TBI every year worldwide, and it is one of the leading causes of disability. Although around 85% of TBI are classified with mild, the remaining TBI patients can have long-term disability. It is estimated that 3.2–5.3 million people are currently living with these consequences [1,9]. There are an estimated 2.87 million TBI cases requiring an emergency department visit every year in the US, and about 43% of these cases go on to have long term disability. About 500,000 children per year in the US have TBI requiring an emergency department visit. These children are under the age of 14 yrs, with the majority being 0–4 years old. TBI can make up about 15% of sports related injuries, and children aged 6 and older are at highest risk for TBI resulting from a sport. Males are more likely than females, at all ages, to sustain TBI [9].

### Underlying molecular and cellular mechanisms

**Figure 1:**
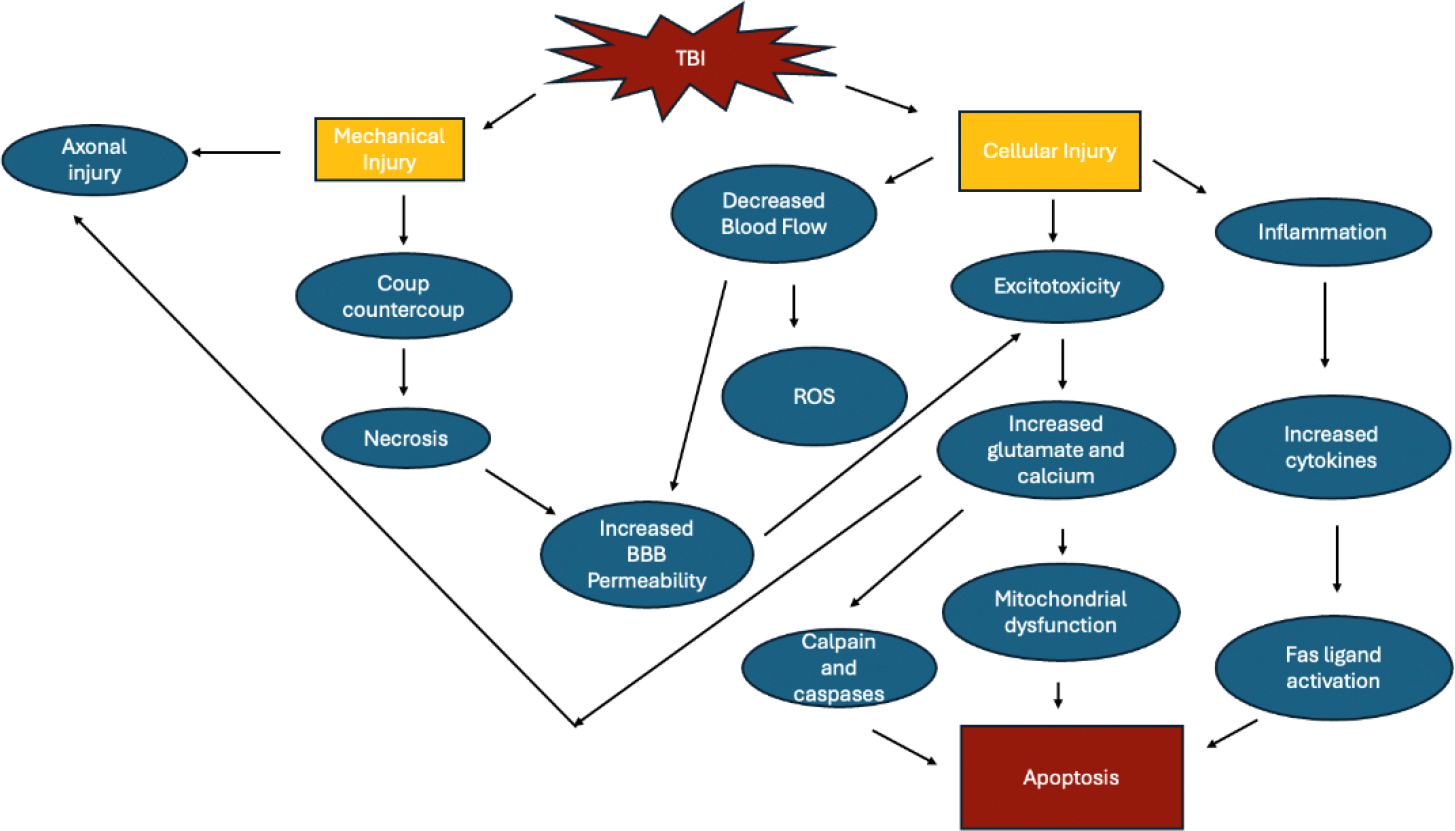
Schematic diagram of the mechanism of traumatic brain injury.

TBI can result in temporary or permanent damage to the neural tissue through both primary and secondary injury. The initial injury triggers a cascade of events that result in inflammation, excitotoxicity, and oxidative stress (Figure 1). Prostaglandins, cytokines and reactive oxygen species are all activated which increases the permeability of the blood brain barrier (BBB), resulting in further damage. Other mediators, such as glutamate and calcium also lead to excitotoxicity and apoptosis of neurons [14]. Mechanical impact to the head can cause damage directly to the brain tissue, resulting in necrosis of the neurons and glial cells in the area. In addition, an area opposite to the site of the blow can have damage due to rebound strikes of the brain to the skull. This is known as coup-contrecoup injury [7]. This type of acceleration-deceleration injury can also lead to diffuse axonal injury (DAI) due to the shearing and stretching of axons. Diffuse axonal injury causes disruption to the cytoskeleton membrane. In addition to mechanical injury, axonal injury can also be a result of proteolysis due to the influx of calcium that occurs in TBI, resulting in damage to the myelin sheath [15].

Restricted blood flow leads to the production of free radicals due to decreased oxygen and energy supply [16]. Although the brain can normally detoxify small amounts these free radicals, the amount produced during TBI overwhelms the system, leading to significant damage. This is due to the high lipid and low antioxidant content of the brain. Ischemia can also result in the production of reactive oxygen species (ROS) and nitric oxide (NO) leading to oxidation in several cellular processes resulting in the formation of the superoxide anion and hydrogen peroxide. These products can go on to cause further ischemia, damage, and necrosis to surrounding structures. Peroxidation of lipids can produce aldehydes which are toxic to ion channels and cytoskeleton proteins. In addition, phospholipase A2 is activated during ischemia which further leads to ROS through arachidonic acid [7]. In addition to these pathways, neuroinflammation can also be triggered. It has been shown that inflammatory modulators such as IL-6 and TNF-α were detected 24 hours post-injury in CSF or brain tissue postmortem. The detection of these is further evidence that the BBB is altered in TBI. In addition, these cytokines can also trigger the apoptotic pathway through Fas ligand [15].

Reactive astrocytic response and microglial activation are prominent compensatory mechanisms in TBI. Increased glial fibrillary acidic protein (GFAP) immunostaining has been shown following injury, proving the astrocytic proliferation. This increase in astrocytes is thought to produce an astroglial scar which is protective an promotes recovery of the neuron through signaling pathways (Figure 1). In addition, activation of the astrocytes can also result in excitotoxicity and increased permeability of the BBB. Microglial cells are also activated, which are responsible for the removal of cell debris and toxic substances. These cells, however, also release inflammatory cells which further contributes to the damage to neural tissue observed in TBI [17]. When the BBB becomes permeable, several mechanisms can then occur resulting in secondary injury, one of which being excitotoxicity. Excess neurotransmitters such as glutamate are released which permits the passage of sodium, potassium and calcium into the membranes of the neuron. All of these substances are involved in creating an action potential, and calcium is involved in downstream activation of proteins involved in apoptosis such as calpain and caspases [15]. Mitochondrial dysfunction can also occur because of increased intracellular calcium and reactive oxygen species. Since this can lead to depolarization in the absence of ATP, ATP production slows and causes impairment to the electron transport chain and oxidative phosphorylation [15].

### Current Treatment Guidelines and Rehabilitation

Immediate treatment is centered around patient stabilization, management of elevated intracranial pressure, prevention of secondary brain injury, and seizure prophylaxis. Long-term treatment is centered around an interdisciplinary team that often includes a neurologist, physical therapist, speech therapist, occupational therapist, psychologist and possibly many more. In the immediate phase, several studies have shown differing treatment strategies which have a favorable outcome in treating the increased intracranial pressure (ICP) that can result from TBI. Increased ICP is one of the most impactful factors in TBI, as it poses an immediate threat to stability of the patient. Traditionally, agents such as hypertonic saline and mannitol can be used to decrease ICP based on the hyperosmolar characteristics which creates a gradient and pulls water out of the brain tissue and into the blood. One study looked at short- and long-term outcomes of patients who were given mannitol, hypertonic saline, or both, and found that all three of these strategies are equally efficacious [18]. Temperature control is also important in the immediate phase, as increased temperatures can lead to further brain damage. The optimal temperature was found to be 36°C-37.5°C, with any higher temperatures requiring immediate attention [19]. The early phases of TBI result in several molecular processes that lead to remodeling of the neurocircuits. This remodeling leaves the patient susceptible to seizures, and thus, seizure prophylaxis with antiepileptic drugs is also important in the immediate phase [6].

After patient stabilization, treatment then centers around reducing the complications associated with TBI. This phase of treatment is highly variable as there is no standard protocol, and an overall lack of therapeutic interventions. New developments are constantly being made in this field, as new discoveries are made about the pathophysiology behind brain injury. One field of treatment that is currently being studied is the use of neuromodulation. Neuromodulation uses chemical or electrical stimulants to alter the activity of neurons, often using electric magnetic field (EMF) stimulation. Some methods under current investigation include transcranial magnetic stimulation (TMS), pulsed electric field stimulation (PEMFS), and transcranial direct current stimulation (tDCS) [7]. Neuromodulation is thought to help build new neuronal pathways and increase neuroplasticity as neurons can be hyper- or hypoactive after periods of injury, and the stimulus can lead to alternate signaling pathways [7]. TMS works by providing rapid pulses of electrical current produced by a magnetic field. PEMFS also uses a magnetic field but uses an electric field in conjunction. In tDCS, electrodes are placed on the patient’s scalp to produce a low amplitude electric current. Each of these techniques is non-invasive and have been proven to have minimal side effects to the patient [20]. In addition, EMF has been shown to improve neuronal activity back to near base line in a swine model after TBI [21]. The detection of EMF abnormalities as well as the immediate introduction of EMF and its efficacy has also been demonstrated in the swine model. Stimulation only 20 minutes after injury was shown to have the greatest impact of return to baseline post-injury [22]. Another study was able to show that PEMFS reduced neuronal cell death in neurons exposed to hypoxia by activating a survival kinase cascade as well as recruiting antiapoptotic proteins [23]. Deep brain stimulation (DBS) has been established in the treatment of other neurologic conditions and movement disorders and is now being looked at in relation to TBI treatment. DBS uses electric stimulation of specific parts of the subcortical areas of the brain. Studies have shown functional improvements in independent living and functional capacity of neuronal circuits in long-term studies [24]. Similar to these simulation and radiation strategies, fluorescent nanozymes have also been studied in their efficacy to treat TBI. The fluorescent nanozymes are engineered to emit near-infrared radiation. These nanozymes were shown to have antioxidant activity and prevent ROS signaling pathways associated with inflammation. They also allow for monitoring the status of the BBB, which has been shown to have significant disruption after TBI [25]. Although this treatment modality is still in early phases, several studies have shown promising results and use on human models should be further investigated.

Another field of scientific discovery that may be useful in the treatment of TBI is regenerative medicine and tissue engineering. Surrounding the difficulty of treating TBI is the limitations on the regenerative capacity of neural tissue. Tissue engineering can be used to support neural regeneration by providing stem cells, growth factors, and biomaterials. One study demonstrated that cell-based therapy of stem cell derived extracellular vesicles in a swine model post TBI led to downregulation of inflammation, and an increase in neuron differentiation and survival [26]. Another study used mitochondria derived from human umbilical stem cells as a treatment in rat models after TBI. As discussed previously, mitochondrial dysfunction is a significant contributor to the pathophysiology and lasting effects after brain injury. By introducing new mitochondrial cells, the density of neurons undergoing apoptosis decreased, there was reduces astrogliosis, and improved sensorimotor function [27]. Preservation of brain architecture as well as neurologic functioning was demonstrated using human umbilical cord derived mesenchymal stem cells in rats after TBI [28]. The use of tissue engineering and regenerative in animal models provides a hopeful new modality of treatment when facing the pathophysiology associated with TBI.

Neuroinflammation is one of the greatest challenges after TBI, and recent developments have suggested addressing lymphatic pathways for relief of symptoms. Manual lymphatic techniques such as craniofacial manual lymphatic drainage have been shown to decrease head pain after moderate injury [29]. In addition, a hydrogel aimed at enhancing lymphatic drainage was developed and studied, which shows promising neuroprotective effects especially when combined with the previously discussed extracellular vesicle therapy [30]. Long-term rehabilitation after TBI is centered around functional ability in addition to attempting to damper the harmful neuronal mechanisms. Returning to work after TBI has proven to be one of the difficulties with this injury, thus a multidisciplinary approach is important to help patients return not only to activities of daily living, but their livelihood as well. Compensatory cognitive training with supported employment (CCT-SE) has been shown to be an important part of this multidisciplinary approach. CCT aims to teach compensatory strategies for long term symptoms of TBI including headache and fatigue. Supported employment provides individualized support directly at the patients’ workplace. Receiving CCT-SE has proven to return patients to work earlier than patients only receiving standard therapies such as a psychiatrist, support group, and other outpatient care. Patients who received CCT-SE had a larger number returning to work at 3 months, although return to work at 12 months was like those receiving standard therapies [31]. There are also several non-pharmacological therapies that have been studied in improving TBI symptoms. Exercise, dietary adaptations such as a ketogenic diet, and environmental enrichment have all been shown to be helpful in restoring neurocognition and decreasing symptoms following TBI [24]. Another study outlines how social communication therapy, which aims to address the communication difficulties following TBI, has helped to improve these symptoms leading to helping patients progress closer to their pre-TBI baseline [32].

Although many studies are being conducted on addressing specific cellular and molecular mechanisms that are damaged during brain injury, current treatment mostly centers around addressing each aspect of a patient’s symptomology. Individualized care as well as a multidisciplinary team is necessary when treating these patients.

### Prognosis

Although TBI leaves many patients with long term disability, one study showed that after 12 months, 75% of patients with moderate TBI and 52% of patients with severe injury had a meaningful recovery. However, although many patients can achieve independent living after moderate to severe TBI, this does not take away from the significant disability they can be left with [10]. Due to the nature and complexity of brain injury, prognosis can be difficult to predict. A recent study aimed to help with this, tracking patients with moderate to severe TBI over 12 months to determine which patients regained independence in their activities of daily living. A summary of this study can be found in Table 2. This study measured disability after TBI using both the GOSE and disability rating scale (DRS). The GOSE is currently one of the most widely used scales when determining outcomes of TBI. It is used in many clinical trials as well, to track progression of symptoms and disease. The GOSE is administered by interviewing the patient and asking them about post-injury difficulties in six different domains of life. These domains include independence in and out of the home, work and social functioning, relationship problems and other difficulties affecting daily living. GOSE is scored 1–8 and can be divided into favorable outcomes, scores 4–8, and unfavorable outcomes, scores 1–3 [10]. The DRS is another tool used after TBI that consists of 4 domains including employability, consciousness, cognitive ability and overall functioning. Cognitive ability includes self-care, which is not included in the GOSE. The DRS is scored 0–29, with 0 being no disability and 29 being an extreme vegetative state [10]. Although these tools are important for tracking recovery, they are mostly used as points of time rather than having prognostic value. People with higher GPSE and lower DRS scores tend to recover more independently.

Aside from daily functioning, prognosis should also look at the effect of long-term symptomology following TBI. It is estimated that 70–85% of patients with mild TBI will have complete resolution of symptoms within 3 months [9]. The most common chronic symptoms that are experienced following TBI include headache, memory problems, concentration difficulties and social abilities. Prognosis prediction remains as one of the outstanding challenges with TBI. Recent studies are looking to find biomarkers and other forms of data that can aid in predicting outcome. The glycemic variability is one of these measures that has been looked at. It was shown that patients with a higher glycemic variability are at increased risk for in hospital mortality and poor consciousness outcomes [33]. Another study looked at IL-8, APOE and CRP levels to predict prognosis, which provided more insight and an improvement to current prediction tools based on medical history, physical exam, and GCS alone. This study also recommends using EEG and cerebral oxygen studies in the future to further improve prognostic ability [34].

### Challenges and Outstanding Questions

Challenges of restoring function in patients with TBI include the categorization methods for initial injury. There is a lot of discrepancy in how to assess for severity and predicted long-term outcomes, which is in part due to the lack of quantitative biomarkers. This can often delay treatment and appropriate testing. One example of this is that neurophysiologic testing is often only reserved for patients who experience greater than 3 months of cognitive impairment, but many patients would benefit from this intervention earlier [35]. Another challenge involves pharmacotherapy surrounding TBI. Although there are several medications used in symptomatic management, there are currently no FDA approved medications specifically for this type of injury [36]. Treatment protocols for TBI have yet to be developed, resulting in several variables and unknowns when it comes to long-term therapy. For example, even within one type of treatment TMS, there is varying methods of delivery. This results in varied outcomes and makes it difficult to assess the efficacy of new treatments [20]. In addition, the underlying cellular mechanisms in upcoming therapeutic strategies such as stem cell treatment are poorly defined.^26^ New cellular and EMF therapies have been studied with promising results in animal studies, but there are few studies done on human subjects, once again making it difficult to assess the efficacy of these new treatment strategies.

## Figures and Tables

**Table 1:** Different methods of assessing severity of traumatic brain injury

Severity	GCS	PTA	Imaging	LOC	GOSE
Mild	13–15	<1 day	Normal	0–30 min	-
Moderate	09-Dec	1–7 days	Normal or abnormal	30 min-24 hours	-
Severe	03-Aug	>7 days	Normal or abnormal	>24 hours	-
Favorable Outcome	-	-	-	-	04-Aug
Unfavorable Outcome	-	-	-	-	01-Mar

GCS, Glasgow Coma Scale Score; GOSE, Glascow Outcome Scale-Extended; LOC, Loss of Consciousness; PTA, Post Traumatic Amnesia.

**Table 2:** Summary of prognostic findings in the track-traumatic brain injury study of McCrea and colleagues [10].

	2 weeks		12 months	
	Moderate	Severe	Moderate	Severe
GOSE >4	41%	12.40%	75%	52.40%
DRS 0	-	-	32%	19%
Returned to work without deficits	10%	1%	49%	34%
Independence in the home	39%	10.70%	69%	50.60%
Independence in traveling	37%	11.40%	64%	49.10%

DRS, Disability Rating Scale; GOSE, Glascow Outcome Scale-Extended

## Key Points

- Immediate management of TBI focuses on stabilization, controlling intracranial pressure and seizure prophylaxis.
- Multidisciplinary rehabilitation (neurology, physical, occupational, speech therapy, psychology) is essential for long-term recovery and return to activities of daily living.
- Neuromodulation therapies have shown promising results in enhancing neuroplasticity, reducing cell death, and improving functional outcomes in animal models.
- Regenerative medicine may help to restore neural architecture and function, but evidence is still preclinical.
- Compensatory cognitive training with supported employment is effective in accelerating early return to work by teaching cognitive coping strategies and providing individualized support in the workplace, while social communication therapy addresses interpersonal and communication challenges, aiding reintegration into daily life.
- Manual lymphatic techniques and biomaterials may improve lymphatic drainage and reduce neuroinflammation after TBI.
- Pharmacologic treatment currently targets symptoms, but there are currently no FDA-approved drugs for treating TBI.
- Outcome measurement tools like Glascow Outcome Scale-Extended and Disability Rating Scale help track recovery, but prognostic ability remains limited.
- Most patients with mild to moderate TBI recover to independent living within 12 months, but severe TBI can often lead to long term disability despite rehabilitation.
- Emerging biomarkers may improve prognosis prediction and treatment plans in the future.
